# Prevalence of Mental Health Problems and Associated Risk Factors among Rural-to-Urban Migrant Children in Guangzhou, China

**DOI:** 10.3390/ijerph14111385

**Published:** 2017-11-14

**Authors:** Jun Wang, Ke Liu, Jing Zheng, Jiali Liu, Liming You

**Affiliations:** School of Nursing, Sun Yat-Sen University, 74, Zhongshan 2nd Rd, Guangzhou 510080, China; wangj96@mail2.sysu.edu.cn (J.W.); liuke@mail.sysu.edu.cn (K.L.); zhengj38@mail.sysu.edu.cn (J.Z.); liujiali@mail2.sysu.edu.cn (J.L.)

**Keywords:** migrant children, mental health, risk factors

## Abstract

Rural-to-urban migration, which has achieved a huge scale during China’s economic reform, is a potential risk factor for the mental health of migrant children. To test this hypothesis, this study assessed the mental health status of rural-to-urban migrant children. Guided by Andersen’s behavioral model, the study explored the risk factors associated with mental health. The study recruited 1182 fifth/sixth-grade children from four private and four public primary schools in Guangzhou in 2014 in a descriptive cross-sectional design. Mental health status was measured by the strengths and difficulties questionnaire. Predisposing characteristics including demographics (e.g., age, gender), social structure (e.g., education, occupation) and health beliefs (health attitude) were recorded. Enabling characteristics including family and community resources and the need for health services were analyzed to explore the risk factors. The results indicate that more rural-to-urban migrant children were classified in the abnormal (21.0%) or borderline (18.8%) categories based on the total difficulties scores, the proportions of which were much higher than those of local children (9.8% abnormal, 13.8% borderline). Factors associated with a greater likelihood of mental health problems included single-parent families, seeking health information actively, family income cannot meet basic needs and poor perceived health status. Compared with the local children, the rural-to-urban migrant children had relatively poor mental health, hence monitoring and supporting mental health for rural-urban migrant children is critical.

## 1. Introduction

### 1.1. Rural-to-Urban Migrant Population in China

Over the past nearly four decades, China’s economic reform has given rise to the influx of rural-to-urban migration. This rural-to-urban migration typically follows a pattern of movement from the largely poor, agriculture-dominated rural areas of the western and central inlands of China (e.g., Sichuan, Henan, Anhui, and Hunan provinces) to cities in coastal locations in eastern China (e.g., Beijing, Shanghai, and Guangdong provinces). Migrants move from rural to urban centers in search of better jobs, increased educational opportunities and other kinds of resources. In the 1980s, rural-to-urban migrants were mainly young adults, while family migration has prevailed since the 1990s [1]. There were 35.8 million migrant children in cities of China in 2010, representing a 41% increase over 2005 [2]. This study is based in Guangdong. Guangdong is a coastal developed province located in southern China. With migrants occupying 30% of its total population (32.01 million/108.49 million), Guangdong province accommodates the largest internal migrant population in China [3]. Guangzhou, as the capital city of Guangdong province, is the largest city in southern China and it is among the top three largest urban economies, along with Shanghai and Beijing. By the end of 2015, there were 5.73 million migrants in Guangzhou (who accounted for 42.44% of the population), an increase of 7% compared to 2014 [4]. Therefore, it has been the leading rural migrant destination since the beginning of China’s economic reform [5]. Rural-to-urban migrant children are mainly students aged 7–15 years, whose parents or other guardians move from rural to urban areas for more than half a year [6]. This definition of rural-to-urban migrants is important, as it demonstrates the distinction between international migrants moving from one country to another and urban migrants moving to another urban area within the same country.

However, due to the household registration system (*hukou* system) in China, rural-to-urban migrant families do not have residency status in the cities they move to. They are considered as temporary residents [7]. As a result, migrants with a non-local resident identity, compared with local residents, have an inferior access to social welfare benefits in aspects such as health care, education, employment, housing, and social subsidies [8].

For rural-to-urban migrant children, one barrier created by the *hukou* system is that migrant children lack access to public schools in cities. In these schools, education is funded by the municipal governments mainly for children with local *hukou*. Most rural-to-urban migrant children have to go to private schools colloquially known as migrant children schools. However, these schools are usually underfunded and staffed inadequately compared to the public schools [9]. Another important restriction related to the *hukou* system is that there are currently separate health insurance systems designed for the rural areas where the migrants are from (New Rural Cooperative Medical System, NRCMS) and for urban areas (Urban Residents Basic Health Insurance, URBHI), differing in financing levels and insurance benefits. The rate of reimbursement for the NRCMS is lower and the reimbursement process is more complicated if it is used outside of the residence registration area of the person [10]. In some places, the NRCMS cannot reimburse medical payments they occur outside of the hometowns of the migrant people [11]. Although new policies have been established in some inflow cities to guarantee that school-age migrant children can obtain the same right as local urban children to participate in URBHI [12], due to the fact that migrant parents are not familiar with new policies and some migrant children are under the school age, the rate for migrant children insured in URBHI is still extremely low [12]. Rural-to-urban migrant children, in particular, are less likely to receive preventive care, and have lower rates of physical check-ups, vaccination and follow-up care [13]; when ill, they are less likely to seek medical care. They receive fewer indicated medications and treatments and stay for a shorter time in hospital than local children [14]. These barriers to access health care may have potentially important implications for their physical health, mental health and social well-being.

### 1.2. Mental Health of Rural-to-Urban Migrant Children in China

The World Health Organization (WHO) has noted that mental health is perhaps the most important aspect of one’s health, as it determines one’s attitude towards life, one’s interaction with others, and how one deals with physical and social surroundings [15]. Mental health problems, for purposes of this paper, include both emotional problems, such as depression and anxiety, and behavioral problems, such as conduct disorders and hyperactivity disorders [16]. Patel [17] estimated that more than one in four children and adolescents meet the criteria for at least one mental health problem in all societies. However, many children go undiagnosed or untreated [18]. The use of validated screening instruments is commonly recommended to improve the identification of mental health problems in community settings [19]. Early recognition of mental health problems at the developmental age is preventive, and can reduce the escalation of mental health issues and improve quality of life [20].

Migration and migration-related processes are widely regarded as having an impact on the mental health of children [21]. While the mental health of international immigrant children has drawn increased research attention [22,23,24,25], knowledge of the mental health status of rural-to-urban migrant children in China is limited. Previous research on rural-to-urban migrant children in China has mostly focused on educational issues such as education rights, dropout rates, and educational consequences [26,27]. Only a few studies have been conducted on the mental health of this population. These studies have reported that rural-to-urban migrant children in China are more vulnerable to mental health problems than local children, including social and separation anxiety, depression, loneliness and low self-esteem [28,29,30]. However, Cheung [31], who conducted a study in Guangzhou, came to almost the completely opposite conclusions, finding that migrant children do not necessarily suffer poorer mental health than their local counterparts. The inconclusive findings concerning the mental health status of rural-to-urban migrant children in China indicate the necessity of further studies. Furthermore, studies in China have not systematically assessed and compared the mental health problems of migrant children and local children while taking different types of schools into account. As to the age span of the subjects in previous studies, no studies were found to mainly focus on senior students in primary schools, who are in a critical period of biological change, rapid growth and developmental potential.

### 1.3. Theoretical Framework for Identifying Risk Factors Associated with Mental Health Problems

Previous studies have found that risk factors associated with the mental health of migrant children were individual characteristics (including gender, age, ethnicity, physical health and so on) [32] and family environment (including parental education, social class, employment, and family function and structure) [33,34]. However, previous studies exploring risk factors were seldom guided by a theoretical framework. To better understand the risk factors associated with mental health problems among migrant children in China, this study uses an adaptation of Andersen’s behavioral model [35]. The Andersen behavioral model was originally developed in the 1960s to explain and predict why families and individuals use health services, to inform policy, and to increase access to health care equitably. Without question, greater access to health care services significantly improves a population’s health and reduces inequity through initial visit, and greater focus on prevention and provision of person-focused comprehensive care with greater continuity and coordination [36]. Therefore, in the revised model [35], Andersen highlights that health care service utilization will also influence a population’s health. Since then, the model has been used to guide the examination of predictors associated with various populations’ health, such as mental health status among children from different racial groups in California, USA [37].

In this study, the risk factors for mental health among migrant children may be explained by the three components of Andersen’s behavioral model: predisposing characteristics, enabling characteristics and needs. Predisposing characteristics include demographics, social structure and health beliefs; enabling characteristics include family and community resources; and needs for health services include both perceived needs for health services by the subject and needs assessed by health professionals. Thus, whilst there may be some individuals who are more predisposed to seek health care services, there has to be enabling resources for them to do so [35]. Where predisposing and enabling conditions are present, the use of health care services would nevertheless only be necessary if the individual perceives that there is a need for treatment or if the individual has been evaluated as needing treatment by a clinician [35]. 

Given the above-mentioned background, this study has two objectives. First, it seeks to describe the mental health status of rural-to-urban migrant children in China and compare it with their local counterparts using strength and difficulty questionnaire (SDQ). Second, it explores the risk factors associated with the mental health of rural-to-urban migrant children, using Andersen’s behavioral model to group variables into predisposing characteristics, enabling characteristics and need.

## 2. Materials and Methods

### 2.1. Research Design

This study was a part of a large research project evaluating health disparities (including physical health, mental health, social well-being, health care utilization and health behaviors) among rural-to-urban migrant children in Guangzhou, China. In this study, a cross-sectional study design comparing two population groups: rural-to-urban migrant children and local children was chosen. The study was conducted in October through December 2014 in Guangzhou, China. Seven hundred and thirty-one rural-to-urban migrant children were recruited at four private schools and 451 local children were recruited at four public schools.

### 2.2. Sampling Procedure

A multistage sampling method was employed in this study: (1) four out of eleven districts of Guangzhou were chosen, because both local and migrant populations reside in these districts; (2) one private and one public school were randomly selected from each of the four districts; (3) from each school, two fifth grade and two sixth grade classes were chosen through convenience sampling; (4) all children in selected classes were invited to participate. To be eligible as a participant, migrant children had to have lived in Guangzhou for more than six months without local *hukou*, and local children have to live in Guangzhou with local *hukou*. Students who were absent during the survey were excluded.

### 2.3. Measures

*Mental health* is measured by the Chinese version of the strengths and difficulties questionnaire (SDQ) [38]. The SDQ has been widely used internationally to identify children from the age of four to 17 with mental health problems [39,40,41], and is a tool that can be administered to parents, teachers, and children. In this study, the self-reported version was used. The SDQ can be useful as a screening measure in the community to identify children whose emotions and behavior are of concern and prompt further assessment or intervention [42]. The SDQ offers several advantages over other similar instruments: (1) its brief form may be less burdensome; (2) it assesses both difficulties as well as strengths; (3) it covers a wide range of mental health problems which are common in general childhood and adolescence; and (4) it could be used by non-professional people with low levels of additional training [19]. These characteristics make it ideal for use in the community or schools for screening and early detection of mental health problems in children.

The SDQ consists of five subscales of five items assessing emotional symptoms (e.g., “often seems worried”), conduct problems (e.g., “often accused of lying or cheating”), hyperactivity-inattention (e.g., “often be restless”), peer relationship problems (e.g., “often be bullied by other children”), and pro-social behavior (e.g., “often be nice to other people”). The scale items were selected based on the Diagnostic and Statistical Manual of Mental Disorders [43] and International Classification of Diseases 10th Revision [44] classifications of childhood psychopathology. Questions are asked about behavior in the past six months and responses are based on a three-point Likert scale (“not true”, “somewhat true” and “certainly true”) indicating how much each symptom item applies to the target child [38]. The 25 items are summed to create a “total difficulty score”. This score can also be classified as normal (low risk of mental health problems), borderline (possibly at risk of mental health problems), and abnormal (at risk of mental health problems) [38]. In previous studies, good internal consistency was evident with a Cronbach’s alpha of 0.79 on self-reported scores [45]. The Cronbach’s alpha of SDQ in this study was 0.72.

*Predisposing characteristics* are personal characteristics that exist prior to the onset of an illness, including demographic, social structure and health beliefs [46]. Demographic factors such as age and gender represent biological imperatives suggesting the likelihood of people’s need for health services [47]. Traditional measures used to assess social structure include education, occupation, and so on [35]. Health beliefs are attitudes, values, and the knowledge that people have about health and health services [35]. Thus, in this study, predisposing characteristics were measured by gender (male/female), age (continuous variable), *hukou* status (migrant children/local children), place of birth (Guangzhou/other place), number of children in the family (one-child family/non one-child family), parents’ employment status (employed/unemployed) and education (primary school or below/junior middle school/senior middle school/college/university or above), family structure (two-parent family/single-parent family), health beliefs (seeking health information actively/not seeking health information actively).

*Enabling characteristics* are those things that make it possible for a person to receive health services which could influence a population’s health, including family and community resources [46]. Family resources include the financial resources at an individual’s disposal to pay for health services [35]. Community resources entail whether an individual has a regular source of care and the nature of that source [35]. In prior research, the most commonly used variable in this category was income and health insurance [46]. In the current study we attempted to capture these by examining three categorical variables: perceived financial status (able to meet basic needs/just to meet basic needs/cannot meet basic needs), housing status (self-owned housing/non self-owned housing) and health insurance status (insured/uninsured). One other community resource variable, distance to the nearest community health center (continuous variable), was also included.

*Need* is how people view their own general health and functional status, as well as how they experience symptoms of illness, pain, and worries about their health and whether or not they judge their problems to be of sufficient importance and magnitude to seek professional help [35]. In this study, we measured need using perceived health status (excellent/good/fair/poor).

### 2.4. Data Collection

The researchers who collected data were graduate students from the University. The study was conducted at the convenience of the school. The children were reminded that their participation in this study was voluntary. After giving instructions, 45 min were allotted for the children to complete the questionnaires including SDQ scales, predisposing characteristics (demographic factors and health belief) and need (most children completed the questionnaire in 20~30 min). Upon completion of the questionnaire individually, researchers checked the questionnaires to ensure that there were no missing items. After that, the children took the parent questionnaire home including predisposing characteristics (social structure) and enabling characteristics, and researchers collected them the day after. All the surveys were coded according to school, grade and class, and there was no identifying information on the returned surveys to ensure anonymity.

For SDQ, Goodman suggests that cases be included only when a minimum of three answers were given on any single subscale [45]. The cases with multiple choices or conflicting options would not be included. The final sample sizes were thus 802 for migrants and 540 for locals. Seven hundred and thirty-one valid migrant responses were received, for a valid response rate of 91.1%. Four hundred and fifty-one local responses were received, for a valid response rate of 83.5%.

### 2.5. Ethical Considerations

The study was approved by the Ethics Committee of the School of Nursing, Sun Yat-sen University (2014ZSLYEC-030). Participants were surveyed voluntarily and anonymously. In the questionnaire instruction, both the parents and children were informed that if they finished and returned the questionnaire to the researcher, it meant they agreed to participate in this survey. Two researchers undertook surveys with a class with 40~50 students, so even if they met face-to-face with the children, these primary school children were still strangers to them. The collected data were stored in a safe and were coded before analysis.

### 2.6. Data Analysis

First, descriptive statistics of the frequencies and means of the predisposing characteristics, enabling characteristics and needs were computed. Then, a Chi-square test (for categorical variables) or *t*-test (for continuous variables) was employed to examine the differences in the above three groups of variables between migrant children and local children. Second, in order to identify participants who were mentally healthy and unhealthy, the raw scores of the five subscales and total difficulties scores of SDQ were grouped into three categories (i.e., normal, borderline, and abnormal). Then, the Chi-square test was used to figure out the differences in mental health between migrant children and local children. Finally, univariate analyses were performed to estimate the significant predictors of mental health problems (total difficulties scores) of migrant children and local children separately. Then, logistic regression was performed for migrant children and local children separately in order to identify the different predictors of mental health problems after controlling their specific characteristics. The dependent variable was the total difficulty scores while the independent variables were screened by univariate analysis. A *p* value of <0.2 derived from the univariate analysis was entered into the model. Hosmer and Lemeshow’s goodness-of-fit (HL-GOF) tests were used to examine the degree of fitness of the models [48]. All statistical analyses were performed using SPSS for Mac 21.0 (IBM Corporation, Armonk, NY, USA).

## 3. Results

### 3.1. Predisposing Characteristics, Enabling Characteristics and Need of the Sample

This study involved 1182 primary school children in the fifth- and sixth-grades, including 731 rural-to-urban migrant children (61.8%) and 451 local children (38.2%). Table 1 shows significant differences in predisposing characteristics, enabling characteristics and need between the migrant and local children (*p* < 0.01), except mother’s employment status (*p* > 0.05). For the predisposing characteristics, compared with local children, there were more boys among the migrant children. The average age of the migrants was older than the locals. About 89.8% of local children were born in Guangzhou, while only 29.8% of the migrants were born in Guangzhou. More than 90.0% of migrants were not the only child in the family, while more than three-quarters (76.7%) of local children were from a one-child family. More of the migrant fathers were employed, and both of the fathers and mothers had lower educational levels than the local parents (*p* < 0.01). More migrant children came from a single-parent family (*p* < 0.05). Compared with local children, migrants were less likely to seek health information actively.

For the enabling characteristics, more of the migrant families reported financial status as “cannot meet basic needs” or “just to meet basic needs” than the local families (73.4% vs. 59.4%). Compared with local families (83.1%), very few migrant families (5.7%) owned their houses in Guangzhou. Only 77.3% of migrant children had health insurance. The average distance to the nearest community health center of the migrant families was far away than the local families. For need, 24.4% of migrant children evaluated their health as excellent compared to that was 34.9% in local children.

### 3.2. Mental Health of Migrant Children and Local Children

Table 2 shows that 60.2% of rural-to-urban migrant children were within normal range on the total difficulties scores for SDQ; 18.8% of them were scored in the borderline category and 21.0% were scored in the abnormal category. The proportion of migrant children within the category of abnormal was more than twice as large as the proportion of local children (9.8%) falling into the same category. With regard to the SDQ subscales, the percentage of problems for migrant children ranged between 54.2% (peer relationship problem) and 8.6% (hyperactivity-inattention). Migrants reported significantly higher rates of abnormal on all five subscales (*p* < 0.05).

### 3.3. Risk Factors for Mental Health Outcomes

The logistic regression modeling was performed to examine the relationship of the predisposing characteristics, enabling characteristics and need variables and mental health problems (total difficulties scores) of the sample.

For rural-to-urban migrant children, in the univariate analysis, gender, parents’ education, family structure, health beliefs, perceived financial status, health insurance status, and perceived health status were identified as risk factors for higher likelihood of mental health problems. Then, these variables, as independent variables, were included in multivariate logistic regression analysis. The final model of the logistic regression showed that family structure, health beliefs, perceived financial status and perceived health status were the major risk factors for mental health problems. Table 3 shows that the migrant children who came from a single-parent family (OR = 3.132) and the children who are not seeking health information actively (OR = 1.860) were more likely to have mental health problems. Compared with children whose family income was able to meet basic needs, the children whose family income cannot meet basic needs (OR = 2.610) were more likely to have mental health problems. Besides, compared with children evaluating their perceived health status as excellent, children evaluating their perceived health status as poor (OR = 6.217) were more likely to have mental health problems.

For local children, in the univariate analysis, health beliefs and perceived health status were identified as risk factors for higher likelihood of mental health problems. The final model of the logistic regression showed that only perceived health status was the major risk factor for mental health problems. Compared with children evaluating their perceived health status as excellent, children evaluating their perceived health status as poor (OR = 5.444) were more likely to have mental health problems.

## 4. Discussion

### 4.1. The Prevalence of Mental Health Problems in Rural-to-Urban Migrant Children

One of the main findings of our study was that, compared to those of local children, mental health problems were more prevalent in rural-to-urban migrant children. More migrant children were classified in the abnormal (21.0%) or borderline (18.8%) categories on the total difficulty scores. Those proportions were much higher than for local children (9.8% abnormal, 13.8% borderline). The differences were significant (*p* < 0.001). This disparity between migrants and locals with regard to mental health was consistent with the previous studies conducted in Guangzhou [49] and Wuhan [50]. Compared to the normative data for SDQ for local children in Changsha [51] and Shanghai [52], the percentages of migrant children in the borderline and abnormal categories for the SDQ were also much larger. This could partly be explained by the fact that migration not only means moving away from some family members and friends and the sense of home, but also the need to adapt to a new cultural environment that involves different sets of moral values and standards. This may have an impact on some migrant children and lead to elevated symptoms of mental health problems [30]. Moreover, given the fact that local children generally enjoy a higher social status, family and community resources, one would expect them to have better mental health [53]. Overall the disparity of mental health problem prevalence among migrant and local children in this study was higher and the disparity was larger than other findings [54]. One possible explanation could be that all migrant children in this study were from private migrant schools which had a mainly middle to low socioeconomic status population, while the previous studies make no distinction among migrant children coming from different kinds of schools. Another possible reason may be that only fifth- and sixth- graders were recruited in this study. They were facing the stress of entering a higher school, which may give rise to many mental health problems.

Significant differences were found in all five SDQ subscales between migrants and locals. The peer relationship problems had the highest prevalence (54.2%) in migrants, compared to 46.7% in locals, which was consistent with previous research [55]. This may be related to migrant families’ high mobility and uncertainty. Most migrant workers had no stable jobs, nor fixed working places [56]. When parents switched jobs, migrant children had to change schools [56]. There was no doubt that it was difficult for students to build a good peer relationship. On the other hand, differences in the values and patterns of behavior between the migrant children and local children (e.g., heavy regional accent, out-of-date clothing, and communication difficulties) might lead to social discrimination that contributes to poor peer relationships. This study also showed that the largest disparity between migrant children and local children was pro-social behaviors such as helping, sharing and caring. Shi [57] found a positive correlation between poor peer relationships and poor pro-social behaviors. One possible explanation might be that it was difficult for migrant children to integrate into urban society when they feel uncomfortable with their peer relationships. Further research would be needed to confirm that.

The results of this study confirmed the use of SDQ as a screening instrument, which distinguished a significant difference for mental health problems between migrant children and local children. Many previous studies proved that SDQ was valid and reliable for the general population [58,59]. European studies conducted by Goodman [19] on the use of the SDQ as an instrument for the screening of mental health problems in an ample sample of the population have underlined a close correlation between elevated scores in the questionnaire and a clinical diagnosis for psychiatric pathologies. Therefore, the findings of this study suggested that the SDQ could potentially be considered as a preliminary screening tool to improve the detection of children’s mental health problems for further assessment.

### 4.2. The Risk Factors Associated with Mental Health Problems

The application of the Andersen behavioral model revealed how predisposing characteristics, enabling characteristics and need among migrant and local children influenced their mental health status.

#### 4.2.1. Predisposing Characteristics

This study showed that being a single-parent family and not seeking health information actively were significant predictors of poor mental health status for migrant children. The association found between single-parent families and migrant children’s poor mental health seemed to be consistent with the previous studies which suggested that residing with a single parent could represent a risk factor for mental health [60]. According to the “China Family Development Report 2014” [61], because of the high mobility, the divorce rate has been getting higher in recent years in migrant families. In our study, 9% of migrant children came from a single-parent family, compared to 5.9% for local children. The stressful life experiences including economic decline and adaptive challenges associated with divorce could interfere with the mental health of both parents and children [60]. Two parents could provide support to each other, especially in their child rearing, as well as multiple role models and increased resources, supervision, and involvement for their children. Therefore, it was commonly assumed that two biological parents provide the optimal family environment for healthy child development and that any deviation from this family structure, such as single-parent family, was problematic for children [62,63].

For health beliefs, this study showed that not seeking health information actively had negative effects on mental health for migrant children. One possible explanation might be that not seeking health information actively represents a negative health attitude influencing emotional responses to health and guiding health-related behavior [64]. It was negatively linked to preventive health behavior, and was posited to potentially harm child functioning including mental health, school performance, and so on [65,66]. Besides, previous studies have reported that, compared to local children, migrant children had lower levels of health information and more health risk behaviors [67,68]. Overall, this finding suggested interventions that act to encourage initiatives in seeking health information. Not only could migrant children acquire more health information, but such interventions may also act to arrest the negative health attitudes leading to mental health problems.

#### 4.2.2. Enabling Characteristics

Migrant children from low income families were more likely to develop mental health problems. Evidence from migrant children in China also demonstrated that low family income was a risk factor [69]. After migration to the city, though migrants got better economic opportunities, their family financial status was still lower than local families [70]. Low family income may pose a mental health threat in the following ways: it leads to less opportunity to acquire education and access to quality health care and more possibilities to unhealthy behavior namely smoking or unhealthy diet [71]; it was a frustrating and stressful experience that had the potential to lead to more mental health problems and illness [72]. Furthermore, it was found that migrant families may stay poor and become part of a chronically impoverished [73] and persistent poverty had stronger negative effects on children’s internalized problems [74], and externalized behaviors [75] than occasional poverty. There was a need for more longitudinal research exploring the migrant family’s income changes.

#### 4.2.3. Need

In this study, poor perceived health was negatively associated with mental health for both migrant and local children. Perceived health status was used in most school-based or community-based studies as a measured variable for the perceived need for health services [46]. Previous studies found that perceived health status was a reliable, valid and simple indicator of mental functioning among children and adolescents in the research [76]. Longitudinal studies among children have concluded that perceived health status was best understood as an enduring self-concept [77]. The finding in this study suggested that school healthcare workers and general practitioners should consider a child’s perceived health status when evaluating their mental health, and when developing interventions and management strategies to improve mental health.

For local children, the results showed that only need was associated with mental health problems. The predisposing characteristics and enabling characteristics were unrelated to mental health problems. The impact of predisposing characteristics and enabling characteristics on mental health problems for local children might decrease due to the significant advantage in social status and family and community resources compared to migrant children. Further research would be needed to confirm that.

### 4.3. Strengths and Limitations

Containing a large sample of fifth- and sixth- graders in primary schools in a typical urban setting in China was one of the strengths of this study. All the rural-to-urban migrant children were from private migrant schools, which are mainly a middle to low socio-economic status population. The findings of this study suggested that the SDQ could potentially be considered as a preliminary screening tool to improve the detection of children’s mental health problems for further assessment. Moreover, this study systematically explored and analyzed the risk factors associated with mental health problems in migrant children in China, guided by Andersen’s behavioral model.

The main limitation of the study was the cross-sectional design, which did not allow us to infer causal relationships, and these should be investigated in a longitudinal study in the future. Mental health problems were more conventionally referred to by indicators such as levels of anxiety and depression, however, the above indicators were not what SDQ measured. “Need for health services” was a variable which could be assessed both by the subjects and by health professionals. In this study, the need was only assessed by the subjects, while the need assessed by health professionals was not feasible because of the large sample size.

## 5. Conclusions

In this study we described the mental health status of rural-to-urban migrant children and compared it with their local counterparts. In order to analyze the multiple factors which may influence mental health in a systematic way, we applied the theoretical framework developed by Andersen, which distinguishes predisposing characteristics, enabling characteristics and need. This study suggested that compared with the local children, the mental health of the rural-to-urban migrant children was relatively poor and needed more attention, and the socio-economic conditions of the migrant family and poor health beliefs or perceived health status might contribute to the occurrence of mental health problems in rural-to-urban migrant children. Future preventative interventions should seek to increase public attention to the mental health status of rural-to-urban migrant children and promote their mental health.

## Figures and Tables

**Table 1:** Predisposing characteristics, enabling characteristics and need of the participants (*n* = 1182).

	Migrant Children (*n* = 731)		Local Children (*n* = 451)		*df*	χ^2^/*t*	*p*
	*n*	%	*n*	%			
**Predisposing characteristics**							
Female	308	42.2	230	51.1	1	8.572 ^1^	0.003
Age (year)	11.04 ± 0.037		10.85 ± 0.034		4	28.005 ^1^	<0.001
9~	19	2.7	0	0			
10~	197	28.0	151	33.7			
11~	279	39.7	213	47.5			
12~	162	23.0	84	18.8			
13~14	46	6.6	0	0			
Place of birth							
Born in Guangzhou	191	29.8	397	89.8	1	378.183 ^1^	<0.001
Not born in Guangzhou ^3^	450	70.2	47	10.6			
One-child family	68	9.3	345	76.7	1	552.136 ^1^	<0.001
Parents employment status (father/mother)					1	10.957 ^1^/0.958 ^1^	0.001/0.328
Employed	678/556	98.4/80.9	416/353	94.8/83.5			
Unemployed	11/131	1.6/19.1	23/70	5.2/16.5			
Parents educational level (father/mother)					4	380.024 ^1^/464.844 ^1^	0.001/<0.001
Primary school or below	154/231	21.8/33.0	12/11	2.7/2.5			
Junior middle school	345/339	48.7/48.4	82/84	18.6/19.0			
Senior middle school	161/102	22.7/14.6	112/134	25.5/30.6			
College	31/17	4.4./2.4	87/104	19.8/23.6			
University or above	17/11	2.4/1.6	147/108	33.4/24.5			
Family structure					1	12.971 ^1^	0.011
Two-parent family	626	91.0	413	94.1			
Single-parent family	62	9.0	26	5.9			
Seeking health information actively	444	66.4	344	78.9	1	19.654 ^1^	<0.001
**Enabling characteristics**							
Perceived financial status					2	27.490 ^1^	<0.001
Able to meet basic needs	162	26.5	164	40.6			
Just to meet basic needs	354	57.9	207	51.2			
Cannot meet basic needs	95	15.5	33	8.2			
Self-owned housing	42	5.7	375	83.1	1	728.477 ^1^	<0.001
Health Insurance	510	77.3	406	96.4	1	71.515 ^1^	<0.001
Distances to the nearest community health center (km)	2.65 ± 7.356		1.79 ± 2.023		1016	6.976 ^2^	0.008
**Need**							
Perceived Health Status					3	22.576 ^1^	<0.001
Excellent	176	24.4	155	34.9			
Good	337	46.7	206	46.4			
Fair	187	25.9	75	16.9			
Poor	22	3.0	8	1.8			

^1^ Differences in proportions: χ^2^-test; ^2^ differences in means: *t*-test; ^3^ Among 450 migrant children, 41.9% of them came from other areas within Guangdong province, 58.1% of them came from other provinces (e.g., Hunan, Jiangxi, and Guangxi province).

**Table 2:** Prevalence of mental health problems in the migrant and local children.

	Migrant Children		Local Children		*df*	χ^2^	*p*
	*n*	%	*n*	%			
Total difficulties scores					2	31.967	<0.001
Normal (0–13)	349	60.2	320	76.4			
Borderline (14–16)	109	18.8	58	13.8			
Abnormal (17–40)	122	21.0	41	9.8			
Peer relationship problem					2	8.045	0.018
Normal (0–2)	135	20.1	117	26.7			
Borderline (3)	172	25.6	117	26.7			
Abnormal (4–10)	364	54.2	205	46.7			
Conduct problem					2	22.519	<0.001
Normal (0–2)	394	58.2	308	69.8			
Borderline (3)	104	15.4	68	15.4			
Abnormal (4–10)	179	26.4	65	14.7			
Emotional symptom					2	8.657	0.013
Normal (0–3)	473	69.6	339	77.6			
Borderline (4)	88	12.9	43	9.8			
Abnormal (5–10)	119	17.5	55	12.6			
Pro-social behavior					2	27.627	<0.001
Normal (6–10)	469	69.5	355	81.8			
Borderline (5)	100	14.8	53	12.2			
Abnormal (0–4)	106	15.7	26	6.0			
Hyperactivity-inattention					2	11.514	0.003
Normal (0–5)	562	83.0	393	89.7			
Borderline (6)	57	8.4	28	6.4			
Abnormal (7–10)	58	8.6	17	3.9			

**Table 3:** Risk factors associated with mental health problems for migrant children in the multivariate logistic regression model ^1^.

	*B*	*S.E.*	*Wald*	*p*	*OR*	95% CI	
						Min	Max
**Predisposing characteristics**							
Single-parent family	1.142	0.403	8.040	0.005	3.132	1.423	6.894
Not seeking health information actively	0.620	0.223	7.724	0.005	1.860	1.201	2.880
**Enabling characteristics**							
Financial status (Able to meet basic needs as reference)			7.776	0.020			
Just to meet basic needs	0.422	0.263	2.570	0.109	1.524	0.912	2.553
Cannot meet basic needs	0.959	0.344	7.760	0.005	2.610	1.329	5.125
**Need**							
Perceived health status (Excellent as reference)			8.473	0.037			
Good	0.114	0.273	0.176	0.675	1.121	0.657	1.914
Fair	0.606	0.305	3.930	0.047	1.832	1.007	3.334
Poor	1.827	0.868	4.431	0.035	6.217	1.134	34.075
Constant	−1.479	0.299	24.413	0.001	0.228		

^1^ The total difficulty score of SDQ in the borderline or abnormal category were defined as mental health problems.
